# Role and Timing of Hematopoietic Cell Transplantation for Myelodysplastic Syndrome

**DOI:** 10.4084/MJHID.2010.019

**Published:** 2010-08-05

**Authors:** Teresa Field, Claudio Anasetti.

**Affiliations:** Department of Blood and Marrow Transplantation, H. Lee Moffitt Cancer Center and Research Institute. Tampa, Florida, USA

## Abstract

Allogeneic hematopoietic cell transplantation (HCT) is the only curative treatment for patients with myelodysplastic syndromes (MDS). Most patients with MDS are older than 60 years and age-associated morbidities limit the patients’ options for curative transplant therapy. Since the development of conditioning regimens with reduced toxicity, the age limitations for HCT have waned for those patients with good performance status. This review will discuss the role of HCT for MDS based on prognostic features, the optimal timing of HCT, and outcomes based on patient age.

## Introduction:

Myelodysplastic syndrome (MDS) is a disease of ineffective hematopoiesis with the only potential for cure by hematopoietic cell transplantation (HCT) from a healthy stem cell donor. The majority of patients develop MDS beyond 65 years of age when the incidence of the disease is greater than 15 per 100,000, as opposed to less than 4 per 100,000 in patients younger than 65 years.^1^ The best supportive care for MDS includes hematopoietic growth factors, transfusions and iron chelating therapy. Recently, two DNA-methyltransferase inhibitors (DMTi), 5-azacitidine and decitabine (aza-2’-deoxycytidine), and the immune modulator lenalidomide have been approved by the US Food and Drug Administration for the treatment of MDS.^2^–^4^ Nevertheless, HCT remains the only curative treatment for MDS. Before the development of reduced intensity regimens a decade ago, HCT had rarely been offered to older patients, but its use has since been extended to patients age over 60 years.

## Classification and Prognostic Groups:

Multiple classification and prognostic systems have been developed in an endeavor to predict disease outcomes including leukemia transformation and survival. The French-American-British classification system initially recognized MDS as a clinical entity but has largely been replaced by the world health organization (WHO) classification.^5^ The International Prognostic Scoring System (IPSS) was developed to predict MDS prognosis, including time from diagnosis to acute myelogenous leukemia (AML) progression or death. IPSS incorporates blast percentage, number of cytopenias and cytogenetic risk into a low, intermediate-1, intermediate-2, and high risk categories. For patients with high risk and intermediate-2 (Int-2) disease, the average time from diagnosis to AML is 0.2 and 1.1 years, respectively, and the average time to death is 0.4 and 1.2 years.^6^ A more refined scheme incorporates transfusion requirements, WHO category and cytogenetic risk into the WHO classification-based Prognostic Scoring System (WPSS) which is a time-dependent stratification that allows predictions both at diagnosis, and after disease advances.^7^,^8^ More recently identified prognostic factors include transfusion dependence, pancytopenia, marrow fibrosis, blast clusters and male gender, and these have been incorporated into improved prognostic models.^9^–^12^

## Induction Chemotherapy or Autologous Transplantation:

These classifications and prognostic scoring systems can aid in determining the most realistic treatment goal and care. Whereas patients with low risk features could use less intensive therapies with growth factors and immune modulation, more intensive treatment is required for controlling advanced disease.^13^ High dose chemotherapy alone is effective in obtaining a complete remission in some patients, however, remissions are short and relapses occur early resulting in a 5-year overall survival (OS) at 8%, according to a meta-analysis of clinical trials conducted at the M.D. Anderson in Houston, Texas, USA.^14^

Autologous HCT outcomes have not faired better. The CRIANT trial evaluated high dose induction followed by consolidation chemotherapy followed by continued chemotherapy versus an autologous HCT for patients without an-HLA-identical sibling donor versus allogeneic HCT for those patients with such a donor. More than half of the 341 patients enrolled did not achieve remission with chemotherapy and did not proceed on trial. Among the eligible patients, 50 had a donor and 85 did not do so: the 4-year OS was 45% versus 27%, respectively, significantly better for those with a donor (P= 0.020). Among those without a donor, 34 received an autograft and 38 continued with chemotherapy: their 4-year disease free survival (DFS) was 30% vs. 17%, respectively, not a significant difference (P=0.98).^15^

Al-Ali et al analyzed the European Blood and Marrow Transplantation Group (EBMTG) data, and compared 593 patients with MDS or secondary AML who received autologous HCT in first complete remission (CR1) versus matched unrelated donor (MUD) HCT with untreated disease or in CR1. The authors found 3-year relapse rates (RR) of 62 % for autologous HCT, 30% for untreated MUD and 24% for MUD in CR1, and observed that relapse after autologous HCT continued overtime while relapse after MUD HCT reached a plateau before 3 years. In this analysis, age 20 – 40 years versus >40 years was not a significant factor in outcomes in the allogeneic arm.^16^

## Allogeneic HCT:

Multiple studies were designed to investigate pre-HCT factors to identify which patient would gain the most benefit from allogeneic HCT.

Deeg et al reported that FAB (French American British) disease stage, cytogenetic risk, and IPSS score predict the incidence of relapse after HCT from compatible sibling or unrelated donors treated with a regimen of targeted busulfan and cyclophosphamide. Patients with low risk IPSS had 80% relapse-free survival (RFS) at 3 years versus 29% for high risk patients. The 3-year cumulative incidence of relapse was 0%, 6%, 29% and 42% for MDS with low risk disease, Int-1, Int-2 and High risk, respectively.^17^ Donor matching also influenced survival after HCT. Sibling and matched unrelated transplant recipients had a similar 3-year RFS (56% vs. 59%) but HLA mismatched transplants were associated with decreased RFS (27%). FAB stage, refractory anemia (RA), RA with excess blasts (RAEB) or RAEB in transformation, affected the relapse risk with a 3-year RFS of 68%, 45% or 33%, respectively, after sibling transplants and 70%, 40% or 17%, respectively, after matched unrelated donor transplants. 17

Sierra et al reported international bone marrow transplant registry (IBMTR) data showing a 40% 3-year average RFS with sibling donor transplants, and found that IPSS category and percentage of marrow blasts at time of HCT influenced relapse risk.^18^ The impact of FAB stage was also found by the Castro-Malaspina et al, in a retrospective analysis of unrelated donor transplants facilitated by the national donor marrow program (NMDP). The two year DFS, relapse incidence and transplant related mortality (TRM) were 29%, 14% and 54%, respectively, advanced FAB stage was associated with an increased risk of relapse, and lower FAB stage with increased DFS.^19^

Guardiola et al analyzed associations of donor source with outcome in a multi-center retrospective analysis of 234 sibling HCT in patients with MDS. The authors compared bone marrow (BM) (n=132) with 102 with peripheral blood (PB) donor transplants, and found a 2-year DFS of 50% with PB versus 39% with BM [RR, 0.79; 95% CI, 0.54–1.14, P < .20], a TRM of 36% with PB versus 42% with BM [OR 0.33, 95% CI 0.15–0.73, P<0.001], and a relapse incidence of 13% with PB versus 38% with BM [RR 0.29, 95% CI, 0.13–0.62, P = .20]^20^ Consistent with results of BM versus PBSC prospective trials in patients with high risk malignancy, the Guardiola paper has set the use of PBSC as the preferred stem cell source for allogeneic HCT in the treatment of MDS.^40^

de Witte et al reported that a longer interval from diagnosis to transplant for RA patients is associated with worse survival. They analyzed 374 transplants with RA in the EBMT registry, 191 had data to calculate the IPSS score. Fifty-nine percent were age greater than 50 years, and the majority had PB grafts (84%). The four-year OS, RFS, NRM and RR were 52%, 48%, 37% and 15%, respectively. By multivariate analysis, transplant within 12 months of diagnosis was a favorable factor for survival (HR 1.4, P=0.05).^22^

Cutler et al addressed the optimal timing of MDS patients proceeding to HCT using a decision analysis from Center for the International Blood and Marrow Transplant Research (CIBMTR) registries and the Fred Hutchinson Cancer Center. Adult patients age 18 – 60 years were included. The model found that early transplantation resulted in improved survival for patients with INT-2 and high-risk MDS, but not for those with earlier stage disease. Patients with low risk disease had an estimated life expectancy of 6.51 year when transplanted at diagnosis vs. 7.21 years if transplant occurs at disease progression. As the low risk cohort did not demonstrate an advantage for early HCT, they should wait for HCT until disease progression.^21^ Therefore, while delaying HCT optimizes survival of early MDS patients with indolent disease and a relatively long life expectancy, it paradoxically makes HCT a less effective therapy for those patients with more aggressive disease.

## Pre-Transplant Chemotherapy:

There is an uncertain role of pre-transplant therapies for MDS while patients are waiting for a suitable donor. The studies noted above found MDS risk factors that predicted outcomes of allogeneic HCT. Several institutions have explored the efficacy of chemotherapy before HCT to reduce the MDS risk, with the hope to decrease the rates of relapse and improve HCT outcomes. Nakai et al described the outcomes of 238 MDS patients undergoing a sibling donor HCT: OS at 5 years was similar for patients who received pre-HCT chemotherapy and obtained a remission and those who did not receive prior chemotherapy 57% vs. 54% P=0.81.^23^

Oran et al reported on MDS (n = 30) and AML (n = 82) patients who were given fludarabine-melphalan as conditioning. Twenty-three of the MDS patients received pre-HCT chemotherapy. Results varied with remission status, with 2-year OS of 66% for patients in CR and 23% for those with active disease and circulating blasts. Cumulative incidence of nonrelapse mortality at 2 years was 20% for patients who had CR and 56% for those who did not have CR. The fraction of patients who received chemotherapy and did not proceed to HCT was not reported.^24^

Scott et al examined 150 patients with MDS or AML arising out of MDS. Among the 38 patients who received a non-myeloablative transplant (NMT), 24 had been treated with pre-transplant induction chemotherapy, with 20 achieving CR. At 3 years, OS, DFS, disease progression, and nonrelapse mortality were 27%, 28%, 41%, and 31%, respectively. There were no survival differences between the 112 patients who received standard myeloablative conditioning and the 38 who received a nonmyeloablative conditioning.^25^

The question of whether pretransplant ‘leukemia-type’ induction chemotherapy provides an advantage to MDS patients treated with allogeneic HCT is under investigation through the Allo-MDS, 2x2, phase III, prospective, randomized trial by the EBMTG.

## Pre-Transplant DNA Methyltransferase Inhibitors:

The approval of 5-azacitidine and decitabine for the treatment of MDS has provided an alternative strategy to prevent disease progression in transplant-eligible patients. In a prospective randomized trial, 5-azacitidine prolonged survival compared to standard supportive care in 191 patients with MDS.^2^,^3^ Sixty percent of the patients had a response (complete response in 7%, partial response in 16% and improvement in 37%) as compared to 5% response using the standard supportive care. Median survival was increased (18 months versus 11 months) and there was a decrease in the time to transformation to leukemia (21 months versus 13 months). The drug was well tolerated with the most common treatment related toxicity of myelosuppression and concomitant infections. Due to the survival advantages reported with 5-azaciticine and the fact that it is relatively well tolerated, its use has become widespread. Patients are typically treated until there is no longer a benefit.

Decitabine has also demonstrated efficacy in treating MDS. Kantarjian et al found that 30% or patients responded but there was no significant improvement in survival. Patients with de novo MDS had a median OS of 12.6 versus 9.4 months in those treated versus standard of care (p=ns). Time to progression to AML was decreased from a median of 12.1 versus 7.8 months (P = 0.16).^4^ The reason for the decitabine studies failure to show improved survival over standard of care controls remains unclear.

During the months needed to obtain a donor, patients are at risk of disease progression, transformation to AML, complications of treatment, and to become ineligible for HCT for any of these reasons. As the median time to death for IPSS high risk and intermediate risk MDS is 0.4 and 1.2 years. Therefore, a large fraction of patients with high risk MDS could deteriorate or die while waiting for a suitable donor or for the transplant to be scheduled. DMTi pretransplant could provide a bridge to HCT by controlling MDS and delaying disease progression during the donor search. It is also possible that pre-HCT DMTi render patients ineligible for HCT because of complications. There are currently no data on the proportion of patients that are treated with DMTi that are eligible to proceed to HCT. However, post-transplant outcomes on patients receiving 5-azacitadine or decitabine are available. Field et al compared 54 patients with MDS or CMML receiving pre-HCT 5-azacitidine (n=30) or no 5-azacitidine (n=24) and outcomes were similar. 26 Cogle et al examined 8 patients receiving pre-HCT 5-azacitidine and reported full donor chimerism in both granulocyte and lymphocyte subsets. 27 Two groups have described pre-HCT decitabine effect on HCT. Lubbert et al described 15 patients with MDS or AML and De Padua et al reported on 17 patients with MDS who received pre-transplant decitabine prior to HCT.^28^,^29^ Both groups found no increased toxicity or adverse effects on HCT outcomes.

The answer to the role of DMTi pre-transplant therapy remains under investigation. Because induction chemotherapy can increase the risk of death or prevent patients proceeding to HCT because of toxicities, DMTi’s are an alternative for patients to control the disease while they await HCT.

## Age and Reduced Intensity Conditioning (RIC):

Most patients are diagnosed with MDS when they are older than 60 years. Until the ‘90s, older patients have been excluded from HCT due to intolerable risk of non-relapse mortality. Older patients also have higher risk MDS than younger patients. Recent understanding that donor engraftment does not require myeloablative conditioning and that disease control is in part provided by alloreactive donor immune cells, has allowed designing reduced intensity regimens that are more tolerable, have decreased mortality and have increased the age that HCT can be offered. 17,30,31,38 Several studies focusing on patients with MDS and AML, have reported comparisons of reduced intensity versus conventional preparative regimens and demonstrated comparable disease-free survival (DFS) (39% to 50%). 32,33,34 Reduced intensity regimens tested in those reports include fludarabine, low dose TBI, busulfan or melphalan.

Although the use of reduced intensity conditioning regimens has decreased transplant-related mortality and extended HCT to older and frailer patients, the risk of relapse has increased. Several reports have assessed the increased risk of relapse with the use of reduced intensity conditioning regimens in MDS. Nakamura et al analyzed results of a fludarabine-melphalan regimen in 43 MDS patients, including 15 who had progressed to AML. The two-year OS, DFS, relapse, and transplant-related mortality were 54%, 51%, 16%, and 35%, respectively. Donor source was not a factor in survival, although there was less relapse when unrelated donors were used (P = .02).^35^ Chan et al described a novel regimen of pentostatin, total body irradiation, and photopheresis in 18 patients with MDS. In this study, 2 patients did not engraft and 2 did not achieve initial CR and died of disease progression. One-year OS, DFS, and nonrelapse mortality were 65%, 64%, and 14%, respectively. 36 Kroger et al reported on 12 high-risk MDS patients who were given fludarabine, busulfan, and ATG induction. Two-year OS and DFS were 26% and 12%, respectively, with 4 patients dying in remission. 37 McCune et al reported on the effect of age on HCT in patients with AML (536) and MDS (535) utilizing data from the Center for international Blood and marrow Transplant Research (CIBMTR). Among patients with MDS, age was not associated with survival, relapse or nonrelapse mortality at 2 years. OS was 35% in the cohort aged 55–59, 45% aged 60–64, and 38% greater than 65. 38 Field et al reported on 121 patients with MDS or CMML receiving fludarabine plus a myeloablative dose of targeted busulfan. Eight-nine were older than 50 years and fifteen older than 65 years. There were no significant differences in OS (P=0.7) or NRM (P=0.15), but there was an increase risk of relapse with increasing age (P=0.002). Two-year OS was 49% (<50 years), 47% (50–59 years), 54% (60–64 years) and 38% (>65 years). Use of pre-HCT DMTi or unrelated donors did not affect outcomes.^31^ Utilization of RIC methods permit proceeding to HCT in selected patients up to 75 years of age with no or minimal co-morbidities.

## Finding A Suitable Donor:

Finding a suitable donor before disease progression is a challenge in MDS due to rapid progression in intermediate and high risk disease. On the average, the time from the initial consult for transplant to the donor cell infusion (including the time to identify a donor, obtain insurance approval and complete the pre-transplant evaluation) ranges from 3 to 4 months. If the siblings are not a match, then an unrelated donor search should be initiated. Donor relation, whether sibling or unrelated, has not been a significant factor for survival of patients regardless of age.

Anasetti et al investigated the time it takes to identify an unrelated donor match. The probability of finding an unrelated donor HLA-A, B, C, DRB1 allele match [7 or 8 of 8] is 89% (Caucasians), 77% (Hispanic) and 64% (African-American). No suitable matches were found in 11% (Caucasian), 23% (Hispanic) and 37% (African American). Time to identifying the 1st donor was a median 20 days [11–59, 95% CI]. The time from identification of donor to cell infusion was a median of 81 days [45–199, 95% CI]. 39 Field et al reported that among 255 MDS patients with MDS or CMML evaluated for HCT, 88% found a suitable donor, and 70% with a donor proceeded to HCT.^31^

## Treatment Algorithm:

**Figure 1.**
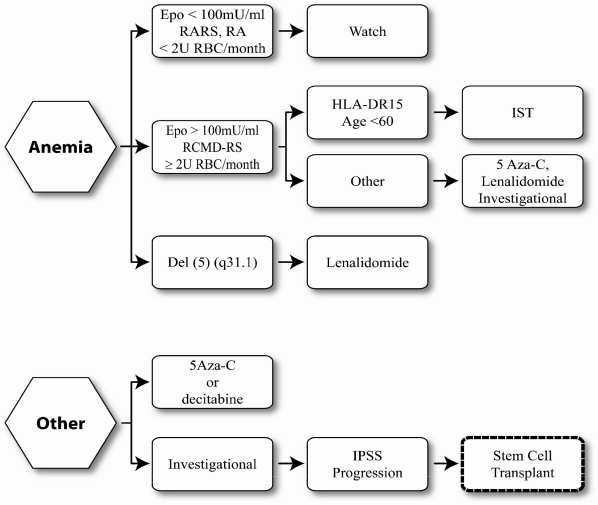
Treatment algorithm for low/int-1 risk MDS (modified from NCCN).

**Figure 2.**
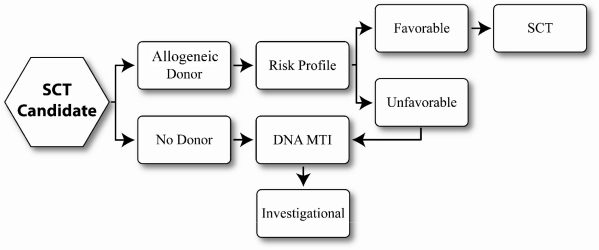
Treatment algorithm for int-2/high risk MDS (modified from NCCN).

**Figure 1 and Figure 2** illustrate treatment decision points in Low risk/Int-1 and Int-2/High risk MDS, according to the National Cancer Center Network MDS consensus panel. For those patients with low risk disease where anemia is the sole unfavorable risk feature and who have low transfusion requirements, watchful waiting is recommended, particularly in those age less than 50 years. Treatment options for those with myeloblasts < 5% but with concomitant adverse features such as increased transfusion needs, severe cytopenias, multi-lineage dysplasia, age greater than 50 years or therapy related MDS include immunosuppressive therapy, hypomethylating agents or enrolling in an investigational trial. Patients with Del (5) (q31.1) should receive lenalidomide. All except those with low risk disease should be HLA-typed and a donor search initiated at diagnosis. At time of progression, HCT can be offered to those with an adequate performance status, if an available donor exists.

Patients with IPSS Int-2 or high risk, who are potential transplant candidates because of adequate organ function and minimal comorbidities should have a donor search initiated at diagnosis and proceed to HCT. DMTi agents can be utilized as a bridge to stabilize the disease until time of HCT. Those without a suitable donor should be offered therapies via an investigational clinical trial.

## Conclusions:

HCT remains the only curative therapy for high risk MDS. Decreased toxicity of the pre-transplant conditioning regimen provides the opportunity to treat suitable patients with allogeneic HCT up to 75 years of age. Treatment with DMTi may allow more patients to reach transplant, however, prevention of post transplant relapse remains a challenge.
